# Conflicting Molecular Hallmarks of Lipedema Reflect Cell-Composition Confounding: A Reanalysis and Germline Reframing

**DOI:** 10.7759/cureus.115591

**Published:** 2026-09-01

**Authors:** Alexandre C Amato, Juliana L Amato, Daniel Benitti

**Affiliations:** 1 Department of Vascular Surgery, Amato Duo, São Paulo, BRA; 2 Department of Gynecology, Amato Duo, São Paulo, BRA; 3 Department of Vascular and Endovascular Surgery, Medical Valens Center, São Paulo, BRA

**Keywords:** bulk rna-seq, cell-type composition, deconvolution, extracellular matrix, gluteofemoral adipose tissue, gwas, lipedema, nlrp3 inflammasome, single nucleus rna sequencing, vegfa

## Abstract

Background: Lipedema is a common, female-predominant disorder characterized by disproportionate, painful lower-limb adipose tissue, and it still lacks an objective biomarker, a disease-modifying therapy, or consensus on whether its core lesion is inflammatory. Its molecular literature is openly contradictory, reporting both immune enrichment and immune down-regulation in the same gluteofemoral depot. Because the dominant morbidity is pain rather than adipose mass, this ambiguity carries direct clinical cost. We therefore asked the following three questions: whether the reported molecular hallmarks survive control for cell-type composition, whether pain and adipose volume behave as separable endpoints, and what the germline architecture implies about the primary lesion.

Materials and methods: From a theory-neutral standpoint, we reprocessed the largest publicly deposited adipose bulk RNA-seq dataset of lipedema (14 women with lipedema versus seven controls, paired by depot) with Salmon (College Park, MD: University of Maryland) and DESeq2 (Seattle, WA: Bioconductor Project), estimated cell-type composition by single-sample gene-set enrichment (ssGSEA), and refitted differential expression with leukocyte and erythroid composition covariates to test identifiability. We also synthesized published treatment cohorts (pain versus volume) and reviewed the architecture of germline genome-wide association studies (GWAS).

Results: There is no transcriptional lipolytic brake (ADRA2A +0.001; lipases unchanged or mildly up). The apparent immune down-regulation is not identifiable from blood content; adjusting for tissue composition reduces genome-wide-significant genes from 1,501 to four, with blood content correlated with disease at r approximately -0.7, so bulk data can neither establish nor exclude immune involvement, including the NLRP3 inflammasome. Pain and adipose volume are dissociable across treatments, so body weight is a confounded endpoint. The germline architecture reported to date (GRB14-COBLL1, VEGFA, RSPO3, ADAMTS9) is adipo-vascular and extracellular-matrix-based rather than immune, although the largest contributing study defined cases by a bioimpedance proxy rather than by clinical diagnosis.

Conclusions: Lipedema's inflammatory status is currently undecidable from composition-confounded bulk tissue rather than settled, which explains the field's contradictory literature; the germline evidence, limited by its case definitions, supports as a hypothesis an adipo-vascular and connective program rather than a primary immune lesion; and pain, not weight, should anchor trials. We specify, but do not perform, the single decisive experiment - paired thigh-versus-abdomen single-nucleus RNA-seq with explicit composition control.

## Introduction

Lipedema is a common, female-predominant condition characterized by disproportionate, painful enlargement of the lower-limb subcutaneous adipose tissue. Despite a prevalence plausibly near 10-12% of adult women, it lacks an objective diagnostic criterion, a dedicated International Classification of Diseases (ICD) code, a validated biomarker, and any disease-modifying drug; the median diagnostic delay exceeds two decades [1]. The field's foundational assumption, that lipedema is fundamentally a disorder of fat, has framed its diagnostics (fat distribution), its outcomes (volume and weight), and its therapeutics (fat removal). More troublingly, its molecular pathophysiology remains not merely unknown but internally contradictory - tissue transcriptomic and proteomic studies have reported both immune enrichment and M2-macrophage enrichment, and immune down-regulation, in the same gluteofemoral depot, and the inflammasome's role is asserted by some and unmeasured by most.

The conventional primary-adipose model also leaves three recurring anomalies unexplained. First, the disease begins and worsens at hormonal transitions (menarche, pregnancy, contraception) yet persists after menopause. Second, the affected fat is metabolically healthier than its mass predicts, with lower HbA1c, higher adiponectin, and a phenotype inversely associated with cardiometabolic and autoimmune disease [2,3]. Third, the dominant morbidity, pain, does not track adipose mass. Lipedema is, moreover, still defined by consensus and exclusion, with an administratively coded prevalence two orders of magnitude below population estimates [4].

This paper does three things, from a deliberately theory-neutral standpoint that invokes no proprietary disease model. Based on replicated germline evidence, we reframe the root of lipedema as an adipo-vascular and connective program rather than a primary immune lesion, without prejudging the presence of a downstream inflammatory component. By reprocessing the largest public adipose RNA-seq dataset, we demonstrate why the tissue literature contradicts itself - a cell-composition confound that renders the disease signal statistically non-identifiable. Finally, we specify the single experiment that would settle it. Throughout, we map what the current data can and cannot support, rather than over-reading a bulk-tissue snapshot into a mechanism.

## Materials and methods

Public data source and sample assignment

We reanalyzed the publicly deposited bulk RNA-seq data from gluteofemoral and abdominal subcutaneous adipose tissue from the lipedema multi-omics study by Straub et al. as follows: 36 paired-end Illumina NovaSeq (San Diego, CA: Illumina, Inc.) runs from 21 women (14 lipedema, 7 controls) with thigh and/or abdominal biopsies [5]. Case and control status was not annotated in the sequencing run table, so we retrieved it from the disease field of each National Center for Biotechnology Information (NCBI) BioSample record (lipedema versus control), and we retrieved the biopsy site (abdomen or thigh) from the sample naming convention. The resulting assignment (lipedema: 10 abdomen and 13 thigh; control: six abdomen and seven thigh) matched the cohort and quality exclusions reported by Straub et al. exactly [5]. Raw sequencing files were downloaded from the European Nucleotide Archive and verified by checksum. The data accession numbers are given in the data and code availability statement. We generated no new tissue, RNA, or sequencing data - biopsy handling, RNA extraction, library preparation, and sequencing were performed by the original investigators and are described in Straub et al. [5]. The deposited libraries we reprocessed were paired-end, with a mean of 24.6 million read pairs per run (range: 19.9-29.5 million).

Transcript quantification and differential expression

Reads were quantified against the GENCODE v44 (Hinxton, England: EMBL-EBI) human reference transcriptome using Salmon (College Park, MD: University of Maryland), with its bias-aware mapping options enabled (mean mapping rate: 84%, range: 82.5-86.0%) [6,7]. Transcript-level counts were summarized to the gene level with tximport (Seattle, WA: Bioconductor Project) [8]. Differential expression between lipedema and control was then tested with DESeq2 (Seattle, WA: Bioconductor Project), including biopsy site (abdomen or thigh) as a covariate so that thigh and abdomen samples were compared on an equal footing [9]. Significance was assessed using a Wald test and Benjamini-Hochberg control of the false discovery rate (FDR), with genes considered significant at an FDR below 0.05. Results are reported per gene as the log_2_ fold-change with its adjusted p-value. The exact software versions, index settings, and statistical model formulas are listed in appendix 1.

Cell-type deconvolution

The relative cell-type composition of each sample was estimated by single-sample gene-set enrichment analysis (ssGSEA) [10], as implemented in the GSVA package (Seattle, WA: Bioconductor Project), using curated marker gene sets for mast cells, M1 and M2 macrophages, total leukocytes, neutrophils, and adipocytes (the adipocyte set serving as a dilution reference) [11]. Differences between lipedema and control for each signature were tested with the Wilcoxon rank-sum test. We chose ssGSEA rather than a reference-based deconvolution method, such as CIBERSORTx, because it needs no external service and returns relative, rather than absolute, cell fractions, which is sufficient to test whether biopsy composition differs between the two groups. Because our claim concerns the collinearity between composition and disease status rather than any absolute cell fraction, we checked the composition difference against three readouts that do not use ssGSEA as follows: the seven-gene erythroid-and-perfusion score described below, whose genes were selected on technical grounds; the raw direction of the contrast, in which about 98% of detected genes are nominally lower in lipedema; and the enrichment of the down-regulated genes for blood, bone marrow, spleen, and thymus signatures reported by the original authors [5]. All three agree with the ssGSEA result. A reference-based method would refine the absolute fractions but could not alter this collinearity, which drives the identifiability result.

Composition-adjusted differential expression (the identifiability test)

To test whether the disease signal could be separated from blood and immune content, we refitted the differential-expression model after adding a per-sample measure of tissue composition. The primary model included each sample's standardized total leukocyte enrichment score as a covariate. As a sensitivity analysis, a second model instead added an erythroid-and-perfusion score, computed from the expression of seven hemoglobin and red-cell genes (HBB, HBA1, HBA2, HBD, ALAS2, SLC4A1, and CA1), to capture technical blood contamination separately from any true biological immune difference. We measured how strongly each composition score tracked disease status using Pearson's correlation, and then compared the number of significant genes and the individual gene results between the adjusted and unadjusted models. To confirm that this adjustment does not remove signal by construction, we repeated the analysis on simulated data with the same design, the same covariate construction, and the same covariate-disease collinearity, under the following two scenarios: true effects that are disease-specific and true effects that act through composition. The script is included in the archived analysis code.

Treatment-cohort extraction (pain versus volume)

For the clinical dissociation of pain and adipose volume, summary outcomes were extracted directly from published treatment cohorts (bariatric surgery, ketogenic or low-carbohydrate diet, lymph-sparing liposuction), recording change in body weight or fat, change in pain (visual analog scale {VAS} or numeric rating scale {NRS}), and any within-cohort weight-versus-pain correlation. This is a narrative synthesis of representative published cohorts rather than a systematic review, and because pain scales (VAS, NRS) differ across studies, effects are compared qualitatively rather than pooled.

Statistical analysis

All differential expression tests used DESeq2 Wald tests with Benjamini-Hochberg FDR control, with significance set at FDR <0.05. Deconvolution scores were compared between groups using the Wilcoxon rank-sum test, and the collinearity between each compositional covariate and disease status was quantified using Pearson's correlation. No data imputation was performed. Analyses were carried out in R (Vienna, Austria: R Foundation for Statistical Computing) using DESeq2, tximport, and GSVA, with transcript quantification by Salmon [7-9,11].

## Results

No transcriptional lipolytic brake in lipedema adipose tissue

We reprocessed the public Straub transcriptome (14 lipedema versus seven controls, paired by depot; quantified with Salmon and analyzed with DESeq2, adjusting for biopsy site; mean mapping 84%) to test whether lipedema tissue was transcriptionally braked at the step of fatty acid release [5]. The robust result was negative, as we found no evidence of a transcriptional lipolytic brake (Figure 1, panel A). The beta-adrenergic receptors (ADRB1: +0.38, ADRB2: +0.40, ADRB3: +0.63) and the lipases (PNPLA2/ATGL: +0.22, MGLL: +0.23, PLIN1: +0.31, PLIN5: +0.50) are, if anything, mildly up, and the alpha-2-adrenergic brake ADRA2A is unchanged (+0.001). The diet-resistance of gluteofemoral fat is therefore better explained by the depot's baseline functional alpha-2-antilipolytic tone and architecture than by lipedema-specific transcriptional lipolytic suppression, consistent with fat mobilizing under a strong caloric deficit [12,13]. Oxidative-phosphorylation genes are up in the unadjusted contrast (NDUFA3: +0.62, COX5A: +0.49, SDHB: +0.42; nominal false-discovery rate {FDR}: <0.05), echoing the differential-expression results reported by Straub et al. and by Santella et al.; however, as shown below, this signal does not survive composition control, so we treat increased oxidative phosphorylation (OXPHOS) as consistent with prior reports rather than newly established here [5,14].

**Figure 1:**
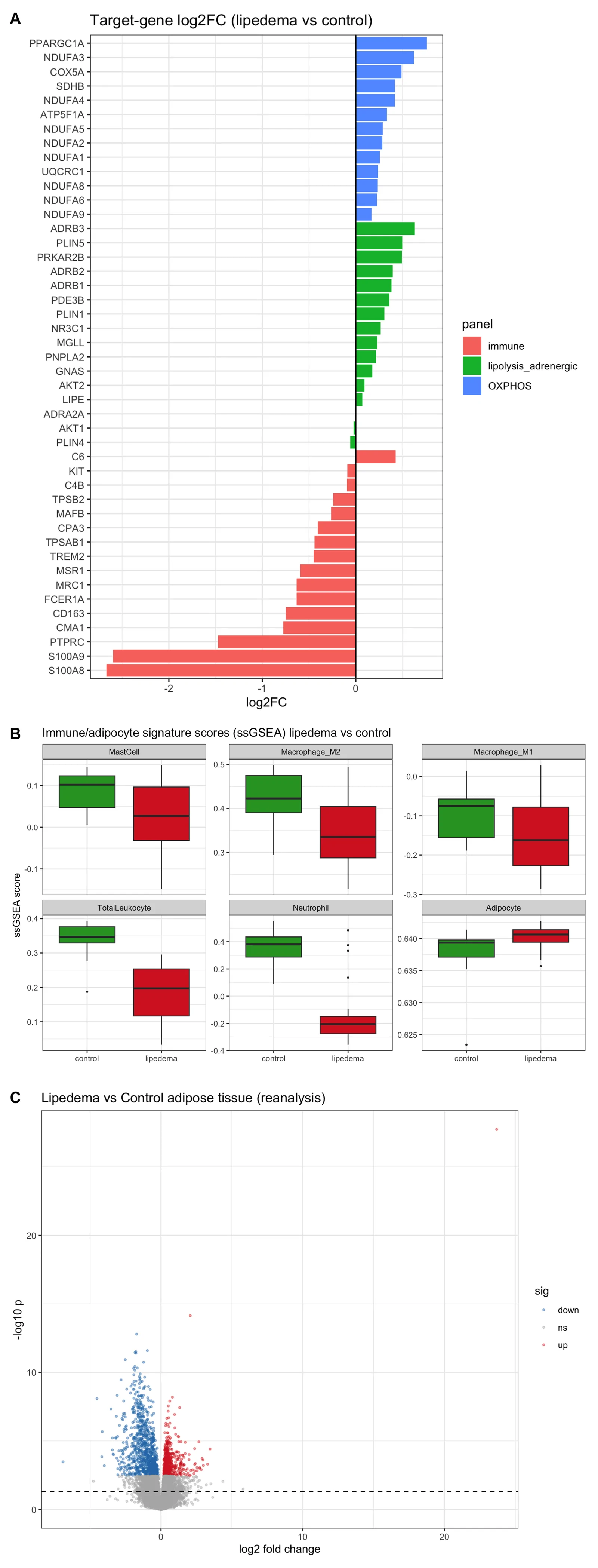
Cell-composition confounding in the lipedema adipose transcriptome. Original re-analysis of public lipedema gluteofemoral RNA-seq (14 lipedema versus seven controls, paired by depot; Salmon to DESeq2 adjusting for biopsy site; mean mapping 84%) [5]. (A) Target-gene log_2_ fold-change - oxidative-phosphorylation genes up (several at FDR <0.05), lipolytic and beta-adrenergic genes up or unchanged (no transcriptional brake; ADRA2A: +0.001), and immune, mast-cell, and M2 markers down (PTPRC at FDR 7×10^-6^; S100A8 and S100A9 at FDR <0.01). (B) ssGSEA cell-type deconvolution - mast-cell (p=0.037) and M2 (p=0.005) fractions and all leukocyte signatures lower in lipedema; adipocyte fraction modestly higher (p=0.009). (C) Composition control - genome-wide significant genes fall from 1,501 (unadjusted) to 29 (erythroid-adjusted) to four (leukocyte-adjusted); blood content correlates with disease at r ≈ -0.7. The transcriptional lipolytic-brake hypothesis is refuted (a robust negative); the immune, inflammasome, and OXPHOS signals are non-identifiable from blood composition, so the apparent immune down-regulation cannot be claimed as disease biology; reduced NLRP3 is the only immune signal that even partially survives erythroid-only adjustment, and it does not survive correction across models. All figures were generated programmatically in Python using the Matplotlib library from the underlying data. FDR: false-discovery rate; OXPHOS: oxidative phosphorylation; ssGSEA: single-sample gene-set enrichment analysis

The disease transcriptome is statistically non-identifiable from cell-type composition

In the unadjusted analysis, the immune compartment looks lower in lipedema. Deconvolution places total leukocyte (p=8×10^-7^), neutrophil, M2 (p=0.005) and mast cells (p=0.037) below control, with the adipocyte fraction slightly higher (Δ: +0.003, p=0.009); at the gene level, PTPRC/CD45 (-1.48, FDR: 7×10⁻^6^), S100A8 and S100A9, and the canonical NLRP3 axis (NLRP3: -1.11, PYCARD/ASC: -0.98, IL1B: -1.96; nominal FDR: <0.05) are all down (Figure 1, panels A, B). On its face, this shows no evidence of active inflammation, inflammasome-driven activity, or mast- or M2-enriched tissue.

This signal cannot, however, be claimed as disease biology, and we state so plainly. Lipedema biopsies have lower blood and leukocyte content than controls, as evidenced by a coordinated collapse of erythroid, platelet, granulocyte, and lymphocyte transcripts (about 98% of detected genes are nominally lower in lipedema). When cell-type composition was added to the model, the entire signal vanished as follows: genome-wide significant genes fall from 1,501 (unadjusted) to 29 (adjusting for erythroid content) to four (adjusting for leukocyte content), and every anchor gene, including VEGFA, IGF1, OXPHOS, the adipocyte program, and the neuroglial pain set, loses significance. The cause is structural collinearity - blood content correlates with disease status at r ≈ -0.7, so "lipedema" and "less blood in the biopsy" are the same statistical axis in this dataset. The data are therefore non-identifiable on the immune axis as follows: they cannot distinguish a true per-cell immune reduction from lower blood content, in either direction. Because the composition covariate is itself derived from the same transcriptome, this adjustment is deliberately conservative and may remove genuine disease signal along with technical contamination. The collapse is not, however, guaranteed by that circularity. Given the observed collinearity, the variance inflation factor for the disease term is about 2, so adjustment widens its standard error by roughly 40%, which demotes borderline genes but cannot, by itself, erase a list of 1,501. In a simulation using the same design, with the covariate constructed in the same way and collinearity fixed at the same value, effects that were genuinely per-cell and disease-specific retained about half of their genome-wide significance (1,576 unadjusted versus 864 adjusted), whereas effects acting through composition retained none. The observed reduction from 1,501 to four matches the second pattern, not the first. The adjustment is also graded rather than absolute: the narrower erythroid score leaves 29 genes, the broader leukocyte score 4, and the survivors are not immune genes. That non-separability is exactly the point, so we read the collapse as non-identifiability rather than as proof of absent biology. Crucially, bulk abundance is not the same as per-cell activity; these data therefore neither support nor exclude an actively inflamed or mast-cell-activated state confined to a patient subgroup, a possibility that only single-nucleus profiling can resolve. The only immune finding that partially survives a technical (erythroid-only) adjustment is reduced NLRP3 (adjusted p=0.045), but even that dissolves under a full leukocyte adjustment. That p-value is genome-wide within its own model, but not across the two composition models we fitted; correcting across those two models makes it non-significant (p=0.09). We therefore treat reduced NLRP3 as a borderline observation consistent with the general non-identifiability, not as a finding that survives it.

Pain and adipose volume are dissociable across treatment cohorts

Across the treatment modalities examined, analgesia and de-bulking dissociate (Table 1, Figure 2). Bariatric surgery removes about 53 kg while leaving pain unchanged and reduces thigh volume by 33% [13,15]. A ketogenic or low-carbohydrate diet reduces pain without a corresponding change in fat mass. In the small LIPODIET cohort, the within-cohort weight-pain correlation was not significant (r=0.28, p=0.46) [16], although, with nine participants, this estimate is too imprecise to establish either an association or its absence. In the larger low-carbohydrate trial by Lundanes et al. (n=70), the reduction in pain was unrelated to systemic inflammation, cytokines, fibrotic markers, or ketosis [17]. The analgesic effect of ketogenic diets is confirmed at the meta-analytic level [18]. Lymph-sparing liposuction abolishes pain with no correlation to aspirated volume (VAS: 80-30); its symptomatic efficacy is likewise established by meta-analysis [19,20]. Quantitative sensory testing localizes small-fiber neuropathy to the thigh, with a normal hand, thereby excluding global central sensitization [21], and with stage-dependent elevations in CGRP and NGF [22]. Morbidity is thus a local tissue property rather than a function of adipose mass, implying that body weight is a confounding surrogate and the wrong primary endpoint for incretin and other pharmacological trials now entering the field.

**Table 1:** Pain and adipose volume are dissociable across treatment modalities. Summary of published lipedema treatment cohorts. Adipose volume responds to caloric deficit and to physical removal, whereas pain responds to tissue removal (durably) and to diet (transiently, by a non-weight, non-inflammatory route) but not to weight loss per se. This is a narrative synthesis of representative cohorts, not a systematic review; pain scales (VAS, NRS) differ across studies and are compared qualitatively. LCHF: low-carbohydrate, high-fat; TNF: tumor necrosis factor; VAS: visual analog scale; LCD: low-carbohydrate diet; NS: not significant; NRS: numeric rating scale

Studies (n)	Intervention	Effect on mass/volume	Effect on pain	Pain versus weight/inflammation
Cornely et al. (2022) [15] (n=13)	Bariatric surgery (prior)	-53 kg (~73% excess-weight loss); BMI: 50 to 32 (p<0.001)	VAS: 7.3 to 7.9 (p=0.28), no change	Pain unchanged despite large weight loss
Fink et al. (2021) [13] (n=31 lipedema, 14 controls)	Bariatric surgery	Thigh volume -33.4% (controls -37.0%; group by time p>0.999)	Volume study; pain not assessed	Lipedema fat mobilizes like control fat
Sørlie et al. (2022), LIPODIET [16] (n=9)	Ketogenic (LCHF), 7 weeks	-4.6 kg (mostly water/lean; fat mass NS: 47.8 to 46.4 kg)	VAS: 4.6 to 2.3 (-50%, p=0.018); returned to 4.2 at week 13	Weight-pain correlation not significant at week 7 (r=0.28, p=0.46), but imprecise with n=9
Lundanes et al. (2025) [17] (n=70)	Low-carbohydrate versus low-fat low-energy diet	LCD lost more fat (-7.0 versus -5.1 kg, p=0.005); LCD lowered hsCRP and TNF-alpha	Pain reduced	No association of pain change with hsCRP, cytokines, fibrotic markers, or ketosis (beta-hydroxybutyrate)
Kruppa et al. (2022) [19] (n=106; 298 procedures)	Lymph-sparing liposuction	Large aspirate; BMI -2.7; durable	Spontaneous pain VAS: 80-30; pressure sensitivity: 80-30 (all p<0.0001)	Durable analgesia, not proportional to tissue removed

**Figure 2:**
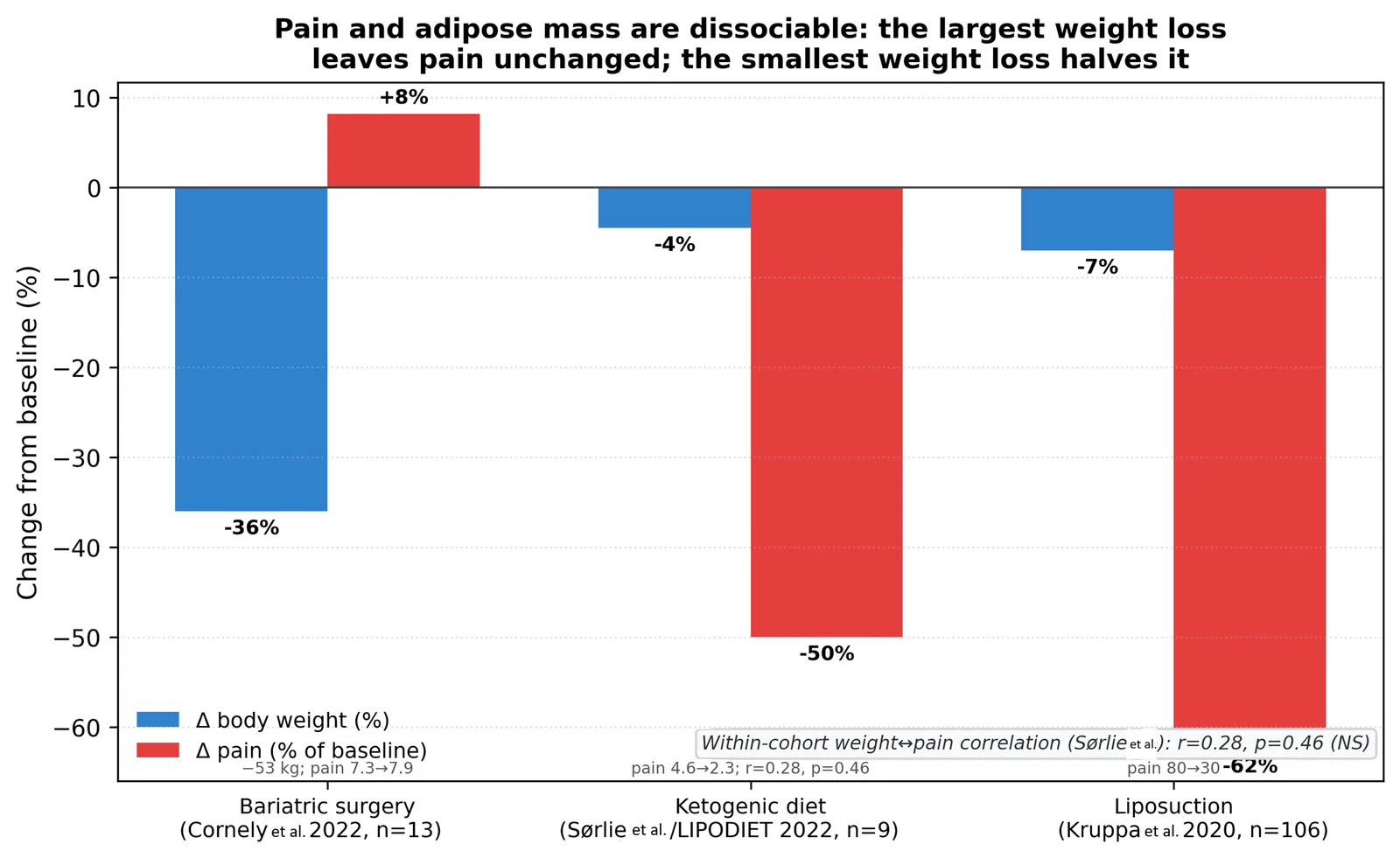
Pain and adipose volume are dissociable across treatment modalities. Per-intervention changes from baseline in body weight and pain, based on published cohort summaries (Table 1). Bariatric surgery produces the largest weight loss yet leaves pain unchanged; ketogenic diets (Sørlie et al., LIPODIET) and lymph-sparing liposuction (Kruppa et al.) produce small changes in weight but roughly halve pain [15,16,19]. Inset: The within-cohort weight-versus-pain correlation is not significant but is imprecisely estimated (r=0.28, p=0.46, n=9) [16]. All figures were generated programmatically in Python using the Matplotlib library from the underlying data.

## Discussion

A germline adipo-vascular and extracellular-matrix reframing

Four independent lines of published evidence point to the germline root of lipedema lying in adipo-vascular and connective-tissue biology, rather than in a primary immune lesion, and none of them exclude a downstream inflammatory component (Figure 3). The first line is genetics. The first genome-wide association study (GWAS) of a lipedema phenotype (18 risk loci, with genetics explaining about 5% of the trait) and a later meta-analysis of clinically diagnosed cohorts overlapping with the UK Biobank both implicate the same genes: GRB14-COBLL1, VEGFA, and ANKRD55-MAP3K1 [23,24]. The largest single signal is RSPO3, a gene in the WNT adipogenesis pathway, and LYPLAL1 and ADAMTS9 (lipid metabolism and extracellular matrix) are also implicated. An earlier GWAS of a UK lipoedema cohort independently supports a heritable basis [25]. These signals concentrate in pathways for cell growth and the cytoskeleton, and in genes expressed in arterial vessels, and they even overlap a varicose vein locus. Crucially, none of the leading loci are immune, inflammasome, or mast-cell genes. These case definitions are, however, imperfect, and the caveat applies to everything that follows - the UK Biobank phenotype was derived from bioimpedance rather than from clinical diagnosis, genetics explain only about 5% of the trait, and no locus survives once pain is added to the case definition. These loci may therefore index favorable lower-body adiposity in general rather than lipedema specifically. Read with that constraint, the inherited architecture is one of favorable lower-body fat distribution together with blood vessels and connective tissue - a hypothesis about the primary lesion that requires testing in clinically ascertained cohorts, not an established fact.

**Figure 3:**
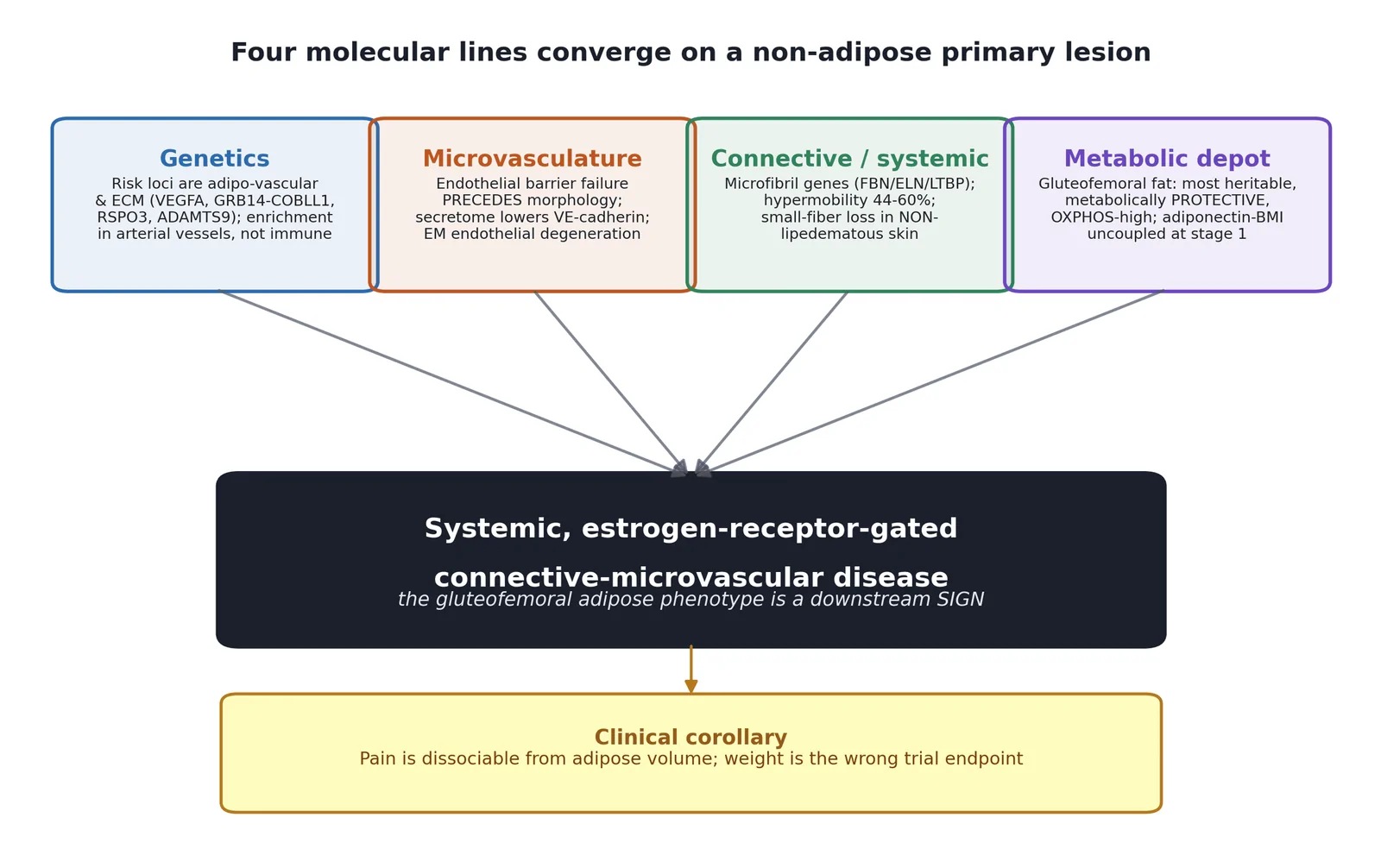
A germline adipo-vascular and connective reframing of lipedema. Four molecular lines of published evidence support this reframing. Genetics (adipo-vascular and ECM risk loci, arterial-vessel enrichment), microvasculature (endothelial barrier failure preceding morphological changes), systemic connective tissue (microfibril genes, hypermobility, extra-limb small-fiber loss), and the metabolic depot (the most heritable, metabolically protective gluteofemoral adipocyte) jointly support, as a hypothesis, a systemic adipo-vascular and connective disorder in which the gluteofemoral adipose phenotype is a downstream sign. The clinical corollary is that pain is dissociable from adipose volume, so body weight is the wrong trial endpoint. All figures were generated programmatically in Python using the Matplotlib library from the underlying data. ECM: extracellular matrix

The second line is the microvasculature. The blood-vessel barrier fails early in lipedema, before any change is visible under the microscope. Fluid released by lipedema stromal-vascular cells is enough, on its own, to loosen the junctions between healthy endothelial cells and make them leakier [26]. Electron microscopy shows degenerating endothelium in affected tissue from women with normal body mass index [27], and capillary fragility is about 3.4 times that of controls and is partly reversible with treatment [28]. The matrix and the vessel are unlikely to fail independently of one another. The elastic microfibrils and basement membrane that invest small vessels also anchor endothelial cells mechanically and sequester latent transforming growth factor beta, so a constitutionally weak matrix leaves the microvasculature poorly supported. The traffic runs in both directions as follows: a leaking endothelium delivers plasma proteins and pro-fibrotic signals into the interstitium, driving stromal remodeling and further matrix stiffening, which in turn alters the mechanical environment of the vessel. The germline signals straddle both compartments rather than either one - VEGFA on the vascular side, ADAMTS9 on the matrix-remodeling side, which is what one expects of a coupled process rather than of two separate lesions. We therefore read the adipo-vascular and connective evidence as a single reciprocal loop, in which the earliest event need not be identifiable in cross-sectional tissue.

The third line is connective tissue. The largest family-based study implicates microfibril and elastin genes (FBN, ELN, LTBP) [29]; joint hypermobility co-occurs in 44-60% of patients [30]; and loss of small dermal nerve fibers is seen even in skin that is not visibly affected [22]. These three observations plausibly share one substrate. Microfibril and elastin genes build the elastic fiber network that gives ligaments and joint capsules their recoil, so germline variation that makes a joint hypermobile is expected to make dermal and subcutaneous connective tissue laxer as well. Small unmyelinated nerve fibers run within that matrix and depend on it for mechanical protection, and small-fiber involvement is likewise described in the hypermobility spectrum disorders that co-occur with lipedema [30]. A systemic matrix defect therefore predicts what is in fact observed - hypermobility, tissue laxity, and loss of small dermal fibers in skin outside the visibly affected limb - which a process confined to the affected fat depot would not, on its own, account for [22]. The following is not exclusive: a constitutionally fragile matrix is the substrate on which any local process, inflammatory or mechanical, would act. This is a testable contrast as follows: it predicts that small-fiber density and matrix architecture should co-vary with hypermobility scores, independently of limb adiposity. The fourth line is the fat depot itself. Gluteofemoral (hip and thigh) fat is the most heritable fat-distribution trait (heritability of about 0.52) and is causally protective against cardiometabolic disease; in lipedema, it maintains this metabolically protected profile despite excess adiposity [2,12,31,32].

Taken together, these lines support a single testable hypothesis that lipedema is the extreme end of the favorable lower-body fat program, expressed on a constitutionally fragile vascular and connective-tissue bed, so that the visible fat is the most obvious sign rather than the essence of the disease. The strong female predominance and the triggering at hormonal transitions fit a mechanism acting through the estrogen receptor, specifically a shift in the balance of the two receptors (more ERβ, less ERα) within adipose stem cells themselves (Bauer et al.), rather than an excess of circulating hormone [33]. A receptor-level mechanism would also explain why the disease persists after menopause. As shown below, however, the tissue-transcriptome support for any receptor model is itself limited by cell composition.

Why the tissue literature cannot converge: composition confounding

Our central result explains why the tissue literature cannot converge. Some studies report immune or M2-macrophage enrichment (Strohmeier et al., using mass cytometry and immunohistochemistry; Streubel et al., in thigh tissue and stratified by disease stage) [26,34]. Others report immune down-regulation (Straub et al. and our own unadjusted reanalysis) [5]. Both kinds of studies measure bulk tissue, and in both, the main feature that separates lipedema from control is the amount of blood and leukocyte content the biopsy contains. In this dataset, that content tracks disease status very closely (correlation r ≈ -0.7). Because the blood-content axis cannot be distinguished from the disease label, the immune signal, including the NLRP3 inflammasome, is non-identifiable - bulk transcriptomics can neither confirm active inflammation nor prove that the immune compartment is depleted. The original study itself points the same way - the genes that were lower in lipedema are enriched for blood, bone marrow, spleen, and thymus signatures (Straub et al.) [5]. That pattern is the expected fingerprint of a biopsy that simply contains less blood, not of an adipose tissue that is intrinsically less immune-active. What was read as reduced immune activity is, on the present reading, reduced blood contamination, and bulk data cannot separate the two. This is not a fault of any single study but a structural limit of bulk adipose tissue, in which variable blood and perfusion content overwhelm the comparatively small disease effect. The long-standing question of whether lipedema is inflamed is therefore, at present, undecidable from the available data. Answering it requires single-cell or single-nucleus profiling, which measures individual nuclei and so largely removes the blood-contamination axis; this makes such profiling necessary rather than optional.

Clinical and translational implications

Three implications follow that do not depend on the unresolved molecular question. The first concerns disease classification. The genetic and connective-tissue evidence supports reclassifying lipedema as a systemic adipo-vascular and connective-tissue disorder, in which the gluteofemoral fat is the visible sign, and supports creating a dedicated ICD diagnostic code; the coded (administrative) prevalence is only about 0.4%, against population estimates of 12-15%. The second concerns diagnosis. Because no single bulk-tissue biomarker is defensible, candidate objective indices, such as a tissue sodium index or an automated dermis-to-subcutaneous ultrasound ratio building on the approach of Amato et al., should be prospectively validated with attention to tissue composition rather than sought in bulk-omics signatures [35]. The third, and most actionable, concern is treatment and trial design. Pain and adipose volume are dissociable, so the primary endpoints of trials should be limb volume and pain, not body weight. Otherwise, trials of incretin drugs (GLP-1 and tirzepatide) and similar agents will report success on weight while leaving the symptom that matters untouched. Because small-fiber neuropathy is localized and is abolished by tissue removal regardless of the volume aspirated, pain is best understood as a property of the tissue itself, to be targeted directly.

A research agenda and the decisive experiment

Several testable inferences emerge from this work, three of which could be addressed today by re-analyzing public data as follows: the dissociation of pain from volume, the non-identifiability of the composition signal, and the way diagnostic delay confounds the apparent link between obesity and lymphedema (appendix 2). The single experiment that would resolve the mechanism is paired thigh-versus-abdomen single-nucleus RNA-seq of clinically defined lipedema, stratified by disease stage and analyzed with explicit control for cell composition. Such a study would settle several questions at once as follows: whether an active mast-cell or type 2 immune program is present in individual cells within a patient subgroup, whether the cell of origin is the adipocyte or the endothelial cell, where the small-fiber pain niche sits, and whether tissue energy metabolism changes with disease stage [27]. Because it measures individual nuclei, it also removes the blood contamination that makes the present bulk data non-identifiable. It is a key missing component of the current evidence base, because the available tissue omics datasets are predominantly bulk.

Limitations

The central limitation is also the key point of this paper: the available tissue transcriptomes are derived from single cohorts, measured in bulk, and confounded by cell composition. Consequently, the disease axis and the blood-content axis cannot be statistically separated (r ≈ -0.7), rendering most apparent molecular findings in either direction non-identifiable. Our own reanalysis shares these limits - 21 participants, a single cohort, a mixture of depots, and no age or menopause covariate available in the public study design. We could not replicate the composition adjustment in an independent bulk dataset, because none suitable is currently public, as follows: the other available bulk adipose RNA-seq of lipedema comprises lipedema participants only, with paired abdominal and femoral biopsies but no non-lipedema comparison group, so a lipedema-versus-control composition-adjusted model cannot be fitted in it. Two points follow from this limitation. The strength of the collinearity we report (r ≈ -0.7) should be read as specific to this dataset until a second case-control cohort is available, since we cannot yet show that lipedema biopsies systematically carry less blood than control biopsies. The structural point, by contrast, does not depend on the sample size as follows: non-identifiability is a property of the design rather than of power, and a larger cohort sampled the same way would estimate both coefficients more precisely without separating them. The germline reframing rests on a GWAS whose cases were defined by a bioimpedance proxy rather than by clinical diagnosis (genetics explaining about 5% of the trait, and no locus surviving once pain is added to the case definition), so it may index favorable lower-body adiposity in general and may not be specific to lipedema. The supporting imaging and omics studies are small and single-center. Our deconvolution used a single relative-abundance method (ssGSEA); a reference-based method would provide an independent robustness check, although the composition difference itself is corroborated by three method-independent readouts. Finally, because the composition adjustment is derived from the same transcriptome it corrects, it is deliberately conservative and cannot separate a technical reduction from a biological one; that inseparability is the non-identifiability we report, not a claim that the biology is absent.

## Conclusions

Our findings support three conclusions. First, the germline architecture reported to date is predominantly adipo-vascular and extracellular matrix rather than immune. Because the largest contributing study defined cases using a bioimpedance proxy, we propose the resulting reframing as a hypothesis to be tested in clinically ascertained cohorts - lipedema may represent the extreme of a cardioprotective gynoid-adiposity program on a fragile vascular-connective-tissue bed, rather than a primary immune disorder of fat, without prejudging a downstream inflammatory component. Second, whether the NLRP3 inflammasome or mast and M2 cells are involved remains unresolved, because the best available tissue datasets are confounded by cell composition to the point of non-identifiability; this limitation may explain the field’s contradictory molecular literature and its stagnation. Third, what remains robust and potentially actionable is narrow but real - there is no transcriptional lipolytic brake, and pain is dissociable from adipose volume, making body weight an inappropriate trial endpoint. Further bulk-tissue snapshots are unlikely to resolve the mechanism on their own. In our view, the most informative next step is composition-controlled, paired single-nucleus RNA sequencing of clinically defined lipedema, ideally with spatial or histological confirmation; other designs that measure individual cells could serve the same purpose. Whatever design is chosen, bulk adipose tissue should be interpreted explicitly in the context of its cellular composition.

## Appendices

Appendix 1

Software, Parameters, and Statistical Models Used in the Re-analysis

This appendix lists the technical specifications of the bioinformatic pipeline. The main text describes each step in plain language; the exact tool versions, index settings, command-line options, and statistical model formulas are collected here for reproducibility. All analysis code and the full derived results tables are archived at Zenodo (Geneva, Switzerland: CERN) and mirrored on GitHub (San Francisco, CA: GitHub, Inc.).

**Table 2:** Software and reference resources. FDR: false-discovery rate

Analysis step	Software/resource	Version	Key settings and parameters
Reference transcriptome	GENCODE (Hinxton, England: EMBL-EBI) (human)	v44	Used to build the Salmon index
Read quantification	Salmon (College Park, MD: University of Maryland)	v1.11.4	Selective alignment against the GENCODE v44 index; k-mer length 31; SSHash index; bias-aware mapping options enabled (equivalent to --gcBias --validateMappings); mean mapping rate 84% (range: 82.5-86.0%)
Transcript-to-gene summarization	tximport (Seattle, WA: Bioconductor Project) (R/Bioconductor)	-	Transcript-level estimates summarized to gene level
Differential expression	DESeq2 (Seattle, WA: Bioconductor Project) (R/Bioconductor)	-	Wald test for the lipedema versus control contrast; Benjamini-Hochberg FDR; significance at FDR <0.05; results as log_2_ fold-change with adjusted p-value
Cell-type deconvolution	GSVA (Seattle, WA: Bioconductor Project) (R/Bioconductor), ssGSEA method	-	Curated marker gene sets (mast cell, M1 macrophage, M2 macrophage, total leukocyte, neutrophil, adipocyte); normalize=TRUE; between-group comparison by Wilcoxon rank-sum test
Statistical environment	R (Vienna, Austria: R Foundation for Statistical Computing)	-	Pearson's correlation was used to quantify covariate versus disease-status collinearity; no data imputation

**Table 3:** Statistical model specifications (DESeq2 {Seattle, WA: Bioconductor Project design} formulas). The design formulas below use standard R model syntax, in which the response is modeled as a function of the terms to the right of the tilde (~). “Site” is the biopsy site covariate; “group” is the disease contrast (lipedema versus control).

Model	Purpose	Design formula	Covariate definition
Baseline differential expression	Primary lipedema versus control comparison, adjusting for depot	~ site + group	site = biopsy site (abdomen or thigh); group = lipedema or control
Leukocyte-adjusted (identifiability test)	Test whether the disease signal is separable from leukocyte content	~ site + leuko + group	leuko = standardized (z-scored) ssGSEA total-leukocyte enrichment score
Erythroid-adjusted (sensitivity analysis)	Isolate technical blood contamination from a biological immune difference	~ site + rbc + group	rbc = standardized (z-scored) erythroid and perfusion score, computed as the mean variance-stabilized expression of HBB, HBA1, HBA2, HBD, ALAS2, SLC4A1, and CA1

Appendix 2

Cross-Domain Inferences and Open Questions From Existing Data

This table is part of the research agenda and is provided as supplementary material. It is deliberately split into (a) inferences that are supported by at least two independent published findings and are, in several cases, testable today by re-analysis of already-public data; and (b) open questions that are plausible on current evidence but require new data to decide, and which we therefore do not assert as findings. “Strength” reflects convergence and directness.

**Table 4:** Inferences supported by existing data, with falsification tests. OXPHOS: oxidative phosphorylation; QST: quantitative sensory testing

#	Inference	Evidence chain (≥2 independent studies)	Strength	Test
1	Diet-resistant fat is hyper-oxidative; the resistance is not a transcriptional lipolytic brake	OXPHOS up in tissue (Straub et al.); low gluteofemoral lipolysis and high alpha2-tone (Alser et al.); thigh fat -33% under bariatric deficit (Fink et al.). This re-analysis (Figure 1, panel A) is as follows: OXPHOS up-confirmed, but beta-adrenergic and lipase genes up and ADRA2A unchanged, so a transcriptional brake is refuted.	Revised (partly established here)	Done (RNA-seq); microdialysis for the functional alpha2 tone
2	Pain and adipose volume are dissociable endpoints	Bariatric -53 kg, pain unchanged (Cornely et al.); diet lowers pain with no accompanying change in fat mass and no relation to inflammation or ketosis (Lundanes et al., n=70; Amato et al., meta-analysis; Sørlie et al., n=9, correlation uninformative); liposuction lowers pain independent of aspirated volume (Kruppa et al.); thigh-restricted small-fiber QST (Dinnendahl et al.)	High	Pooled re-analysis of pain versus weight versus inflammation across published cohorts
3	Diagnostic delay fabricates the obesity-lymphedema association	Early-screen cohorts lower BMI (Amato et al.) versus obese tertiary cohorts; lymphedema risk rises with BMI	High	Compare BMI and lipo-lymphedema distribution: early-screen versus tertiary cohorts
4	The bulk immune signal is non-identifiable from composition; bulk data can neither establish nor exclude mast-cell/M2 involvement or an active per-cell inflammatory state	Bulk immunity down (Straub et al.) versus bulk M2 up (Streubel et al.). This re-analysis: apparent mast-cell (p=0.037), M2 (p=0.005), and leukocyte reductions co-occur with a higher adipocyte fraction (p=0.009) and with the collapse of genome-wide significance under composition adjustment, so bulk abundance cannot separate a true per-cell change from dilution (Figure 1, panels B, C)	Non-identifiable at bulk level	Paired thigh-abdomen single-nucleus RNA-seq (Discussion), to resolve per-cell mast-cell and type 2 activity

**Table 5:** Open questions requiring new data (not asserted as findings).

#	Open question	Supporting observations (not yet decisive)	Data needed to decide
5	Does estrogen act via a receptor-ratio shift (ERβ up, ERα down), local and intracrine rather than via a circulating-ligand excess?	Aromatase down and altered receptor response in lipedema adipose stem cells (Bauer et al.); disease persists after menopause	Tissue versus serum estradiol pre/post-menopause; ERα: ERβ ratio in adipose
6	Is disease “stage” a continuous fluid-and-fibrosis variable rather than a set of discrete grades?	Tissue-sodium imaging and bioimpedance vary monotonically across stages	Stage-stratified mitochondrial omics, BMI-matched per stratum
7	Is the tissue truly edema-free, or do vascular permeability markers move with treatment?	Elevated tissue sodium; macromolecular leak without hyperemia; diet lowers pain and volume while vascular factors shift	Tissue versus plasma VEGFA across treatment; test the endothelial barrier as target rather than anti-VEGF
8	Does a two-axis serum biomarker (lymphatic plus metabolic) outperform any single analyte?	A lymphatic-axis marker (PF4) and a metabolic-axis panel (ceramides and adipokines; Straub et al.) are orthogonal	Measure both axes in existing sera and train a joint classifier
